# Optimization and characterization of immobilized thermostable α-amylase from germinating Sword bean (*Canavalia gladiata* (Jacq.) DC.) seeds on DEAE-cellulose and chitosan bead for operational stability

**DOI:** 10.5511/plantbiotechnology.24.0326a

**Published:** 2024-06-25

**Authors:** Saijai Posoongnoen, Sutthidech Preecharram, Jinda Jandaruang, Theera Thummavongsa

**Affiliations:** 1 Division of Chemistry, Faculty of Science and Technology, Nakhon Ratchasima Rajabhat University; 2 Department of General Science, Faculty of Science and Engineering, Kasetsart University Chalermphrakiat Sakon Nakhon Province Campus; 3 Chemistry Program, Faculty of Science and Technology, Sakon Nakhon Rajabhat University; 4 Division of Biology, Faculty of Science and Technology, Nakhon Ratchasima Rajabhat University

**Keywords:** chitosan bead, DEAE-cellulose, immobilization, Sword bean (*Canavalia gladiata* (Jacq.) DC.) seeds, thermostable α-amylase

## Abstract

Thermostable α-amylase from germinating Sword bean (*Canavalia gladiata* (Jacq.) DC.) seeds has been successfully immobilized on DEAE-cellulose (ICgAmy1) and chitosan bead (ICgAmy2) support materials. Optimum conditions of immobilization for DEAE-cellulose and chitosan bead revealed 97% and 96% immobilization yield, respectively. The optimum pH and temperature of both DEAE-cellulose and chitosan bead immobilized α-amylases were pH 7 and 70°C. Both ICgAmy1 and ICgAmy2 were high stability over a wide pH range of pH 5-9 and a temperature range of 70–90°C. In addition, ICgAmy1 and ICgAmy2 led to an operationally stable biocatalyst with above 74% and 76% residual activity after 10 reuses, respectively. Immobilized α-amylases showed high storage stability with 81% (ICgAmy1) and 85% (ICgAmy2) residual activity after 120 days of storage. The easy immobilization process on low-cost, biodegradable, and renewable support materials exhibited an increase in the enzyme operation range and storage stability which reduces production costs. This makes immobilized amylases an effective biocatalyst in various industrial applications especially a potential candidate for bioethanol production, a key renewable energy source.

## Introduction

Amylases are the most popular and applied enzyme, about 30% of the total global enzyme market. Alpha amylase has been applied in many fields such as medicines, food, paper, textiles, detergents and starch industries, bio-energy production, and chemical analysis etc. (Paul et al. 2021). Alpha amylase (α-amylase, 1,4-α-D-glucanohydrolase, EC 3.2.1.1) acts as a catalyst to break down of random α-1,4 glycosidic bond in polysaccharides such as starch and glycogen, resulting in oligosaccharides and glucose (Farooq et al. 2021). Currently, the industry is focusing on the use of enzymes instead of chemicals because the enzymes can be recycled, specific to reactants and reactions, high efficiency, safe for the environment and consumers, catalyze in mild conditions thus saving energy and raw materials (Jegannathan and Nielsen 2013; Sundarram and Murthy 2014). The main sources of amylase are microorganisms, animals, and plants. In plants, germinating seeds produce amylase, which plays a key role in catalyze the starch deposited in the endosperm resulting in sugar which will be used in the process of plant growth (Xie et al. 2007). Alpha-amylase from germinating Sword bean (*Canavalia gladiata* (Jacq.) DC.) seeds (CgAmy) has been reported an interesting property that can catalyze the reaction at wide pH range from 3.0 to 8.0, high activity at high temperatures in the range of 50–70°C with optimum activity at 70°C (Posoongnoen and Thummavongsa 2020). Amylase from germinating seeds is suitable and a good choice for biotechnology and food applications (Muralikrishna and Nirmala 2005). However, enzymes have limited applications due to their unstable nature and the surrounding environment affects the enzyme. Enzymes may lead to the denaturation in extremely low pH, high temperatures, and in the presence of organic solvents. Thus, enzyme immobilization is the most efficient method to improve enzyme activity and stability. Immobilized enzymes have many advantages, such as high stability and efficiency, reusability, continuous operation, the ease of product purification, more efficient process control, stable quality products and easy to stop the reaction (Yang et al. 2017). Natural organic polymers are highly attractive as material support for immobilization considering their natural abundance, and easy availability (Bilal and Iqbal 2019). Cellulose is a biodegradable biopolymer and has been successfully used to immobilize many enzymes. The immobilized β-galactosidase on DEAE-cellulose had immobilization yield exceeded 90% and maximum galactooligosaccharides yield of 53% (Osman et al. 2014). Chitosan is commonly applied as a material for enzymatic immobilization due to its inexpensive, easy to find, environmentally friendly, non-toxic, biodegradability, and biocompatibility (Verma et al. 2020).

In this research, α-amylase from germinating Sword bean seeds (CgAmy) has been immobilized on DEAE-cellulose and chitosan beads. The immobilization factors were optimized. The properties of immobilized amylases were characterized and compared. Furthermore, the storage stability and reusability of the immobilized amylases were analyzed for further industrial applications.

## Materials and methods

### Enzyme extraction and purification of α-amylase from Sword bean seeds (CgAmy)

Enzyme extraction and purification was performed following the purification method of our previous report (Posoongnoen and Thummavongsa 2020).

### Immobilization of α-amylase on DEAE-cellulose

Diethylaminoethyl (DEAE) cellulose (10 g) was mixed with 200 ml of double distilled water (DDW). The mixture was stirred slowly overnight. Swollen DEAE-cellulose was filtered and then incubated with 200 ml of 0.5 M HCl for 1 h. DEAE-cellulose was filtered and washed with DDW until the pH is 7.0. The solution was mixed with 200 ml of 0.5 M NaOH, stirred for 1 h at 4°C. Then, it was washed with DDW until pH 7.0. DEAE-cellulose was suspended and stored in 0.1 M Phosphate buffer pH 6.0 at 4°C.

The aliquots DEAE-cellulose was mixed with α-amylase (CgAmy) at different amounts of 3, 4, 5, 10, 20 mU and in the presence of soluble starch (0.5% w/v). The mixed solution was stirred overnight at 4°C. The preparation was treated with glutaraldehyde at various concentrations of 0.01, 0.02, 0.05, 0.10 and 0.20% v/v, shaken at 4°C with constant stirring at various time conditions of 1, 2, 3, 4 and 5 h. The ethanolamine was added to a concentration of 0.01% v/v to stop crosslinking and then stand at room temperature for 90 min (Ashraf and Husain 2010).

### Immobilization of α-amylase on chitosan bead

Chitosan powder of 4 g was dissolved in 200 ml of 2% v/v acetic acid solution. The mixture was heated and constantly stirred at 45°C. The solution was added in a syringe. The solution was dropped into 1 M NaOH and stirred constantly to form globular beads. The chitosan beads were stand for 1 h at room temperature and filtered using whatman No. 1 filter paper. The chitosan beads were washed with DDW to a neutral pH and then dried at room temperature for more than 24 h.

The optimum conditions for α-amylase immobilization on chitosan bead were studied. Prepared chitosan beads were activated with glutaraldehyde at various concentration conditions of 0.5, 1.0, 2.0, 3.0, 4.0% v/v under constant stirring at 35°C for 3 h. The activated chitosan beads were washed thoroughly with DDW to remove the excess glutaraldehyde and dried at room temperature. Activated chitosan beads were immobilized with α-amylase at 5, 10, 20, 40, 60 mU to obtain maximum immobilization. The mixed solution was incubated at temperature of 4°C at various times of 2, 4, 6, 8 and 10 h. The unbound protein was removed by washing 4–5 times with 0.1 M Phosphate buffer pH 6.0. The chitosan beads were stored in 0.1 M Phosphate buffer pH 6.0 at 4°C (Weber et al. 2019).

### Immobilization efficiency

Efficiency of α-amylase immobilization on DEAE-cellulose and chitosan bead support materials was calculated by using the following formula. 
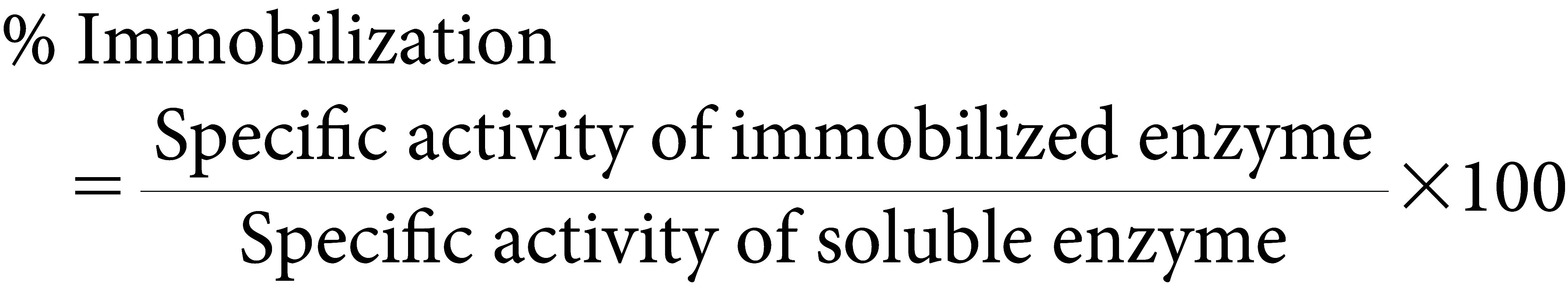

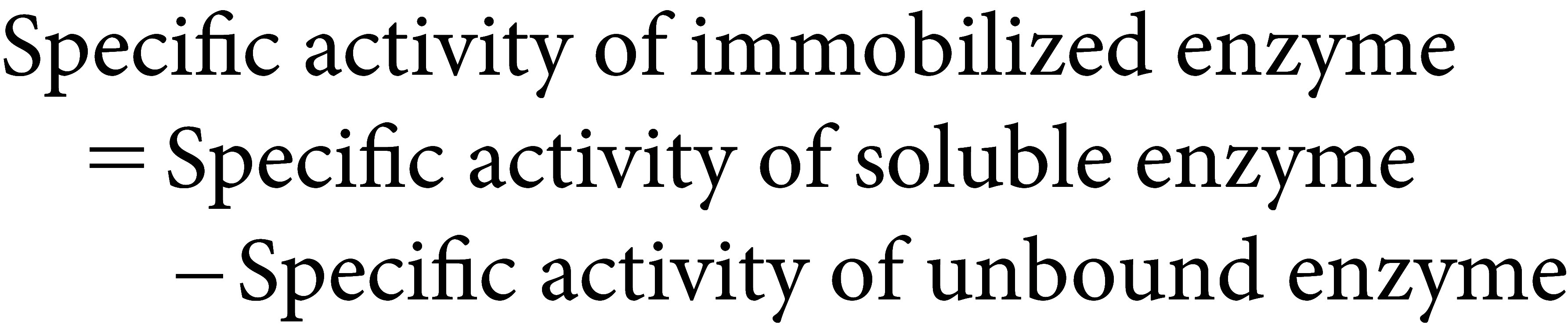


### Characterization of immobilized α-amylase from Sword bean seeds on DEAE-cellulose (ICgAmy1) and chitosan bead (ICgAmy2)

#### Optimum pH and temperature

The immobilized α-amylase ICgAmy1 and ICgAmy2 were studied by Amylase activity assay (Posoongnoen and Thummavongsa 2020) at various pH conditions: 0.1 M Sodium acetate buffer (pH 4.0–5.0); 0.1 M Citrate phosphate buffer (pH 5.0–6.0); 0.1 M Potassium phosphate buffer (pH 6.0–7.0); 0.1 M Tris-HCl buffer (pH 7.0–9.0) at 37.0°C. Enzyme activity was calculated in % Relative activity relative to the pH affected to maximum activity (100% Activity).

The immobilized α-amylase ICgAmy1 and ICgAmy2 were analyzed by Amylase activity assay at various temperature conditions ranging from 30°C to 90°C. The enzyme activity was calculated in % Relative activity by comparing with the temperature affecting high activity (100% Activity).

#### pH and heat stability

The immobilized α-amylase ICgAmy1 and ICgAmy2 were incubated at different pH conditions ranging from pH 4.0 to 9.0 for 60 min at 4°C. Amylase residual activity in % Residual activity was calculated based on the amylase activity at 60 min compared to that at 0 min (100% Activity).

The immobilized α-amylase ICgAmy1 and ICgAmy2 were incubated at various temperature conditions ranging from 30.0 to 90.0°C in 0.1 M Potassium phosphate buffer pH 7.0 and 0.1 M Tris-HCl buffer pH 7.0 for 60 min, respectively. Amylase residual activity was analyzed in % Residual activity, calculated from the activity at 60 min compared to that at 0 min (100% Activity).

#### Kinetic constant

The immobilized α-amylase ICgAmy1 and ICgAmy2 were analyzed by Amylase activity assay at a concentration of 1–10 mg ml^−1^ starch in 0.1 M Tris-HCl buffer pH 7.0 at 70.0°C for 10 min. Lineweaver–Burk plots were done to determined Michaelis–Menten Constant (K_m_) and Maximum velocity (V_max_) of the immobilized enzymes.

#### Storage stability

The immobilized α-amylase ICgAmy1 and ICgAmy2 were stored in optimum pH of 0.1 M Potassium phosphate buffer pH 7.0 and 0.1 M Tris-HCl buffer pH 7.0 at 4°C for 120 days, respectively. The immobilized enzymes were analyzed for residual activity by Amylase activity assay every 30 days.

#### Reusability

The immobilized α-amylase ICgAmy1 and ICgAmy2 were reused 10 times and analyzed % Residual activity using Amylase activity assay. After each amylase activity analysis, the immobilized enzymes were washed with 0.1 M Tris-HCl pH 7.0 and 0.1 M Potassium phosphate buffer pH 7.0, respectively.

### Statistical analysis

Every experiment was analyzed 3 replicates. Experimental results are reported in mean (x̅)±S.E. (Standard error). The results were statistically analyzed by analysis of variance and comparing the mean differences by Duncan’ s Multiple range test method at 95% confidence level (*p*<0.05).

## Results

### Optimum conditions for CgAmy immobilization on DEAE-cellulose (ICgAmy1)

The α-amylase was immobilized on DEAE-cellulose at various conditions to obtain optimum immobilization (Table 1). The % immobilization of 0.01, 0.02, 0.10 and 0.20% v/v glutaraldehyde concentration were 22.58, 42.74, 63.25 and 55.34, respectively. Concentration of 0.05% v/v glutaraldehyde provided the highest immobilization efficiency of 96.15%. The enzyme concentration influenced the immobilization yield, the maximum of enzyme concentration for immobilization was 5 mU. Enzyme concentrations more than 5 mU decreased the enzyme immobilization efficiency to 65.46% (10 mU) and 52.14% (20 mU). The immobilization efficiency of the enzyme was increased with increasing of the incubation time of the enzyme and DEAE-cellulose. Up to 3 h, the enzyme had the highest of an immobilization efficiency of 97.36%. While the immobilization time at 1, 2, 4 and 5 h were decreased of 26.67, 63.97, 60.70 and 61.43% immobilization, respectively. Optimization of immobilization of CgAmy showed that the yield of immobilization was greatly influenced by the concentration of glutaraldehyde, the amount of enzyme and the duration of the immobilization of the enzyme and DEAE-cellulose. Using the optimum conditions for CgAmy immobilization on DEAE-cellulose (ICgAmy1) of activation with 0.05% v/v glutaraldehyde coupled with 5 mU enzymes for 3 h, the immobilization yield was 97.36%.

**Table 1:** Table 1. Optimum conditions for immobilization α-amylase from germinating *Canavalia gladiata* (Jacq.) DC. seeds (CgAmy) on DEAE-cellulose and chitosan bead support materials.

DEAE-cellulose		Chitosan bead	
Conditions	Immobilization (%)	Conditions	Immobilization (%)
Glutaraldehyde concentration (%)^a^			
0.01	22.58	0.5	35.38
0.02	42.74	1.0	70.58
**0.05**	**96.15**	**2.0**	**74.15**
0.10	63.25	3.0	52.58
0.20	55.34	4.0	40.04
Enzyme amount (mU)^b^			
3.0	31.26	5.0	80.03
4.0	48.15	10.0	86.67
**5.0**	**78.35**	**20.0**	**90.15**
10.0	65.46	40.0	88.04
20.0	52.14	60.0	87.80
Incubation time (h)^c^			
1.0	26.67	2.0	86.47
2.0	63.97	4.0	90.46
**3.0**	**97.36**	**6.0**	**95.46**
4.0	60.70	8.0	91.21
5.0	61.43	10.0	85.72

Immobilization conditions. ^a^ Glutaraldehyde concentration: 5 mU enzyme, 3 h (DEAE-cellulose); 20 mU enzyme, 6 h (chitosan bead). ^b^ Enzyme amount: 0.05% glutaraldehyde, 3 h (DEAE-cellulose); 2.0% glutaraldehyde, 6 h (chitosan bead). ^c^ Incubation time: 0.05% glutaraldehyde, 5 mU enzyme (DEAE-cellulose); 2.0% glutaraldehyde, 20 mU enzyme (chitosan bead).

### Optimum conditions for CgAmy immobilization on chitosan bead (ICgAmy2)

The concentration of glutaraldehyde used for immobilization influenced the immobilization yield of the enzyme (Table 1). This research used glutaraldehyde to activate the chitosan bead to achieve covalent bond immobilization between the chitosan bead and α-amylase. When increasing the concentration of glutaraldehyde from 0.5 to 1.0%, the % immobilization increased from 35.38% to 70.58%. The maximum of % immobilization was observed at a concentration of 2.0% v/v glutaraldehyde. At an increasing of more than 2% v/v glutaraldehyde, the immobilization efficiency decreased gradually to 52.58% (3.0% v/v) and 40.04% (4.0% v/v). The results revealed that enzyme concentration affects enzyme immobilization. With increasing enzyme concentration, the immobilization efficiency was also increased, resulting a maximum of % immobilization at 20 mU enzyme (90.15%). At enzyme concentrations of 40 mU and 60 mU, the enzymes had immobilization efficiency of 88.04% and 87.80%, respectively. Optimal immobilization conditions (95.46%) were obtained when incubating the enzyme with chitosan bead at 6 h. While incubation time of 2, 4, 8 and 10 h had % immobilization of 86.47, 90.46, 91.21 and 85.72, respectively. The maximum immobilization efficiency of CgAmy2 was 95.46% by activation with 2% v/v glutaraldehyde and coupled with 20 mU enzymes for 6 h.

### Characteristics of immobilized α-amylase from Sword bean seeds on DEAE-cellulose (ICgAmy1) and chitosan bead (ICgAmy2)

#### Optimum pH and temperature

**Figure 1:**
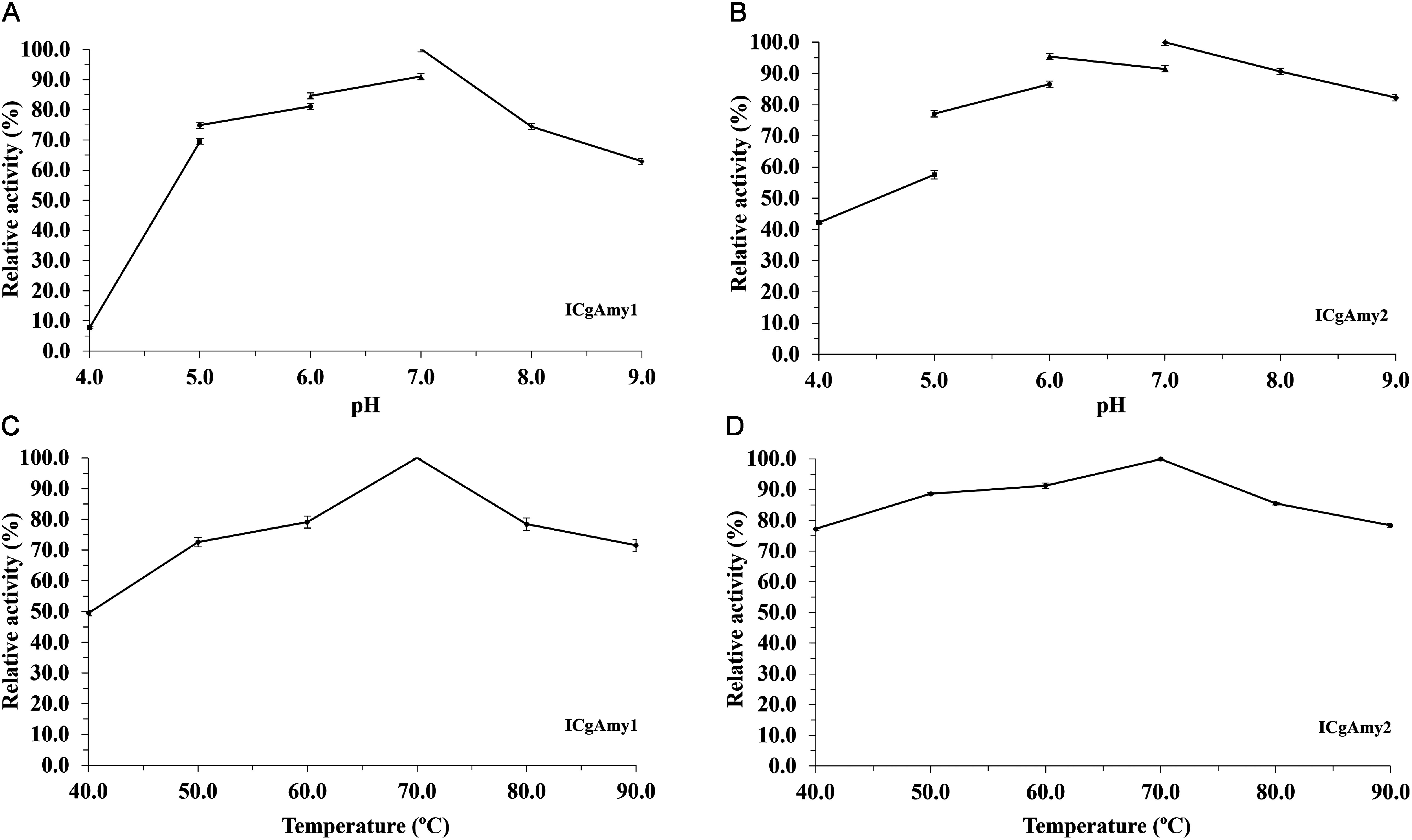
Figure 1. Optimum pH (A) and Optimum temperature (C) for the activity of α-amylase immobilized on DEAE-cellulose support materials (ICgAmy1). Optimum pH (B) and Optimum temperature (D) for the activity of α-amylase immobilized on chitosan bead support materials (ICgAmy2). ICgAmy1: Immobilized α-amylase from germinating Sword bean seeds on DEAE-cellulose; ICgAmy2: Immobilized α-amylase from germinating Sword bean seeds on chitosan beads.

The effect of pH on the immobilized α-amylase activity was shown in Figure 1A, B. The immobilized α-amylase on DEAE-cellulose (ICgAmy1) and chitosan bead (ICgAmy2) have the highest activity at pH 7.0. The chitosan bead immobilized α-amylase was highly active over a wide pH range from pH 5.0 to 9.0 with relative activity >75% (Figure 1B) as well as immobilized α-amylase on DEAE-cellulose with >70% relative activity at wide pH ranges from pH 5.0 to 9.0 (Figure 1A).

Enzyme immobilization on DEAE-cellulose (ICgAmy1), the enzyme catalyzed the maximum activity at 70°C (Figure 1C). At high temperatures of 60, 80 and 90°C, ICgAmy1 was still highly catalyze by 79, 78 and 72% relative activity, respectively. The α-amylase immobilization on chitosan bead showed that the ICgAmy2 was active over a wide temperature range from 40 to 90°C with % relative activity greater than 75%. The optimum temperature of ICgAmy2 was high temperature of 70°C (Figure 1D). The ICgAmy2 was highly catalyzed at 50, 60, 80 and 90°C with % relative activity of 89, 91, 86 and 78, respectively.

#### pH and heat stability

**Figure 2:**
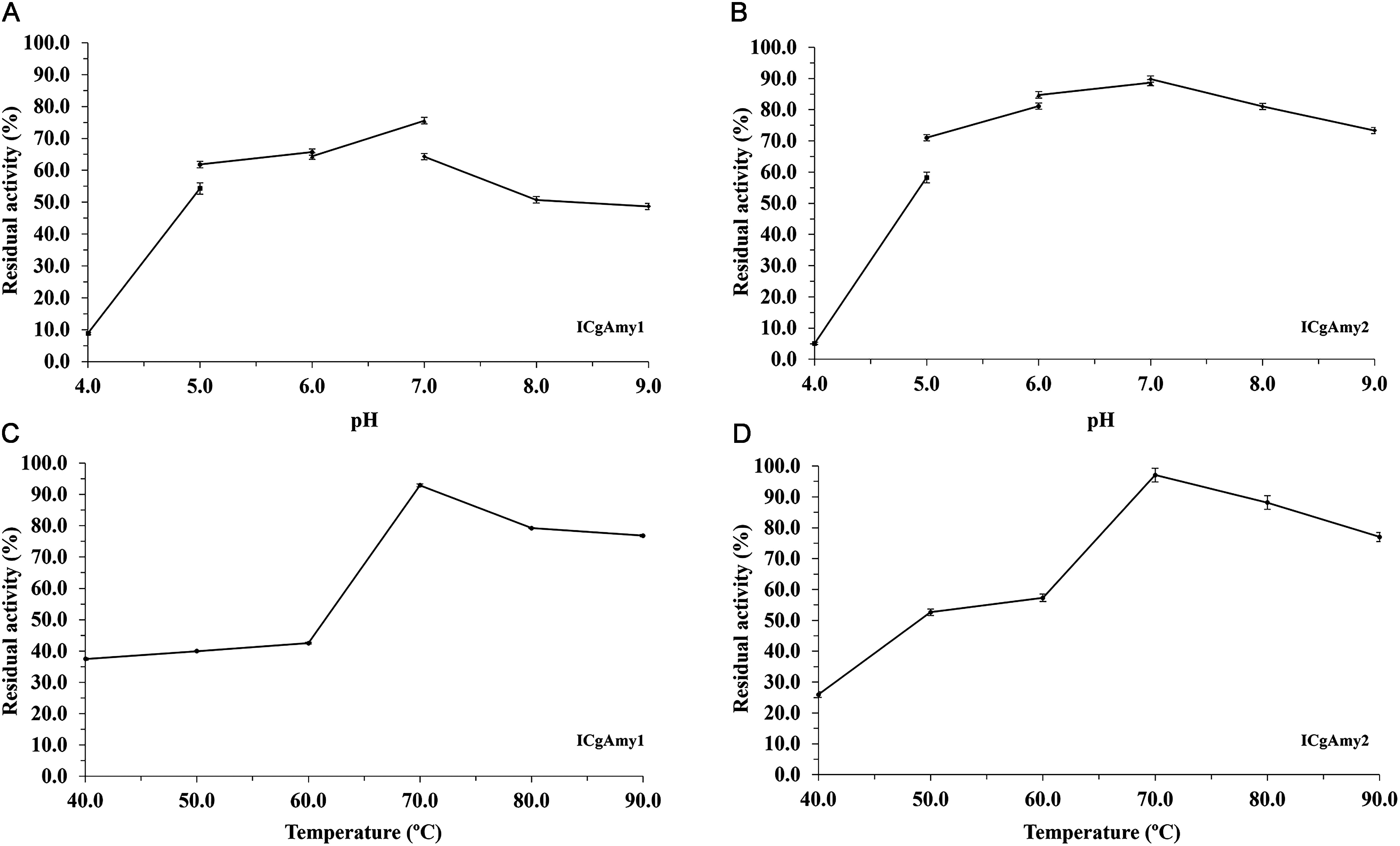
Figure 2. pH stability (A) and Heat stability (C) of α-amylase immobilized on DEAE-cellulose support materials (ICgAmy1). pH stability (B) and Heat stability (D) of α-amylase immobilized on chitosan beads support materials (ICgAmy2). ICgAmy1: Immobilized α-amylase from germinating Sword bean seeds on DEAE-cellulose; ICgAmy2: Immobilized α-amylase from germinating Sword bean seeds on chitosan beads.

The immobilized enzyme on chitosan bead (ICgAmy2) showed high stability (>70% residual activity) over a wide pH range from pH 5 to 9, with maximum stability at pH 7.0 (Figure 2B). The immobilized enzyme on DEAE-cellulose (ICgAmy1) also showed the most stability at pH 7.0. Furthermore, the immobilized enzyme was highly stable over a wide pH range from 5 to 9 with % residual activity >60% for DEAE-cellulose (Figure 2A).

The enzymes immobilized with DEAE-cellulose and chitosan bead showed maximum stability at 70°C (Figure 2C, D). The enzymes were stable over a wide temperature range from 70–90°C with residual activity >75%. The maximum residual activity was up to 97% (ICgAmy2) and 93% (ICgAmy1) after incubation at 70°C for 60 min. At temperatures up to 90°C, α-amylase immobilized on chitosan bead and DEAE-cellulose lost less than 23% of its activity after incubation for 60 min.

#### Kinetic parameter

The kinetic parameters analysis of ICgAmy1 and ICgAmy2 using Lineweaver-Burk plot with starch as substrate were shown in Table 2. The K_m_ values of ICgAmy1 and ICgAmy2 were found to be 5.08 mg ml^−1^ and 5.88 mg ml^−1^, respectively. Our previous report found that the K_m_ value of free α-amylase CgAmy was 3.12 mg ml^−1^ (Posoongnoen and Thummavongsa 2020). The V_max_ value of immobilized α-amylase on the support matrix of DEAE-cellulose and chitosan bead were 0.30 and 0.13 U ml^−1^, respectively.

**Table 2:** Table 2. Kinetic parameters K_m_ and V_max_ of α-amylase immobilized on DEAE-cellulose (ICgAmy1) and chitosan bead (ICgAmy2) compared to free α-amylase (CgAmy).

Enzyme	K_m_ (mg ml^−1^)	V_max_ (U ml^−1^)	Reference
CgAmy	3.12	3.70	Posoongnoen and Thummavongsa 2020
ICgAmy1	5.08	0.30	This study
ICgAmy2	5.88	0.13	This study

#### Reusability

**Figure 3:**
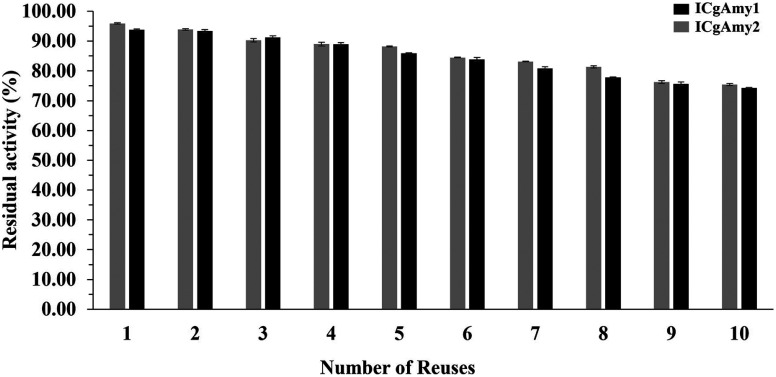
Figure 3. Reusability of α-amylase immobilized on DEAE-cellulose (ICgAmy1, Black) and chitosan bead (ICgAmy2, Grey) support materials for 10 times.

The immobilized α-amylase on DEAE-cellulose (ICAmy1) and chitosan bead (ICAmy2) remained above 74% residual activity after 10 catalysis times (Figure 3) and led to biocatalysts with operational stability. ICAmy1 and ICAmy2 showed 74% and 76% residual activity after 10 reuses, respectively. When the immobilized α-amylase was reused for reactions, the binding strength between the support matrix and the enzyme was decreased. This causes the enzyme to leach from its immobilization with the support matrixs and loss of activity. Moreover, frequent encounters with the substrate in the active site led to distortions resulting in a decrease in catalytic efficiency.

#### Storage stability

**Figure 4:**
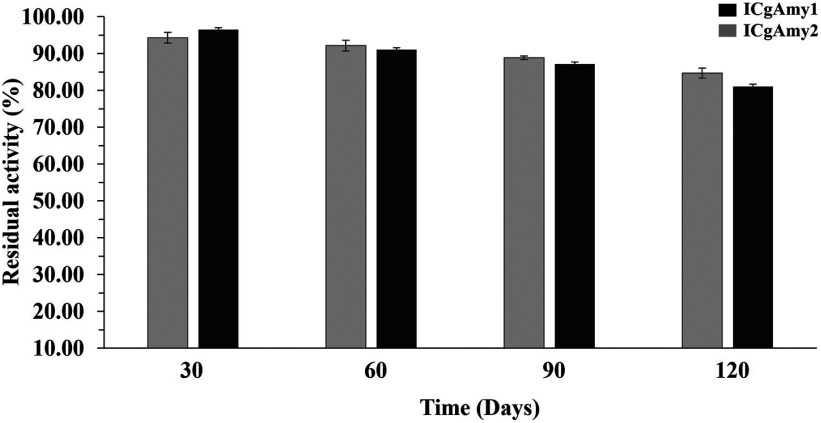
Figure 4. Storage stability of α-amylase immobilized on DEAE-cellulose (ICgAmy1, Black) and chitosan bead (ICgAmy2, Grey) support materials in the buffer of optimum pH at 4°C for 120 days.

The storage stability of immobilized α-amylase on DEAE-cellulose (ICAmy1) and chitosan bead (ICAmy2) after 120 days of storage at 4°C was shown in Figure 4. The loss of α-amylase activity for the immobilized α-amylase on chitosan bead after 120 days of storage at 4°C was 15% activity and compared to the immobilized α-amylase on DEAE-cellulose, the loss of amylase activity was 19% activity over the same period. Loss of enzyme activity during storage is thought to be caused by enzymatic deactivation.

## Discussion

Enzymes are generally expensive, cost of the enzyme account for 15–20% of the cost of production in the case of biofuels (Singh et al. 2019). Highly efficient enzyme immobilization can reduce cost because immobilized enzymes are reusable and have increased stability. In this research, DEAE-cellulose and chitosan bead support materials were chosen for the immobilization of CgAmy, with immobilization efficiency as high as 97.4% and 95.5%, respectively. In comparison with previous research, α-amylase from wheat (*Triticum aestivum*) was also immobilized onto DEAE-cellulose with 86% immobilization under optimal conditions (Singh and Kayastha 2014). In Sword bean, the method of immobilization of CgAmy on DEAE-cellulose was modified, which was different from the previous research. Starch was added in the immobilization step to prevent bonding at the enzyme catalytic site. To stop crosslinking of the immobilization, ethanolamine was added in the final step of immobilization. In addition, CgAmy was also immobilized on a chitosan bead to compare its properties with the immobilized CgAmy on DEAE-cellulose. Immobilization of the raw starch saccharifying amylase on chitin flakes by crosslinking and covalent bonding had an immobilization yield of 62.5% and 55.1%, respectively (Nwagu et al. 2021). The decrease in the immobilization yield at high enzyme loading is due to packing and overlapping of enzyme molecules in pore spaces of the support leading to inaccessibility of the enzyme active site and nonbinding of the enzyme to the substrate (Salazar-Leyva et al. 2016). Immobilization efficiency decreased as the concentration glutaraldehyde more than 2.0% v/v suggesting to aggregation, precipitation, and loss of enzyme activity (Kumari and Kayastha 2011). Low immobilization efficiency of short enzyme immobilization time due to insufficient time of the connection between the enzyme and support materials.

The optimum pH of immobilized α-amylase ICgAmy1 and ICgAmy2 was pH 7.0, while the free α-amylase CgAmy has optimum pH of 6.0 (Posoongnoen and Thummavongsa 2020). The ICgAmy1 and ICgAmy2 exhibit a shift to the base side and have a wide optimum pH range. These results suggest to the covalent binding of the enzyme to the matrix, pH change in bead environment and diffusional limitation. Purified α-amylase from mung bean has 1.4 units shift of pH to the base side after immobilization on chitosan bead (Tripathi et al. 2007). The optimum pH of the free soybean α-amylase was 5.5, while the optimum pH was 8.0 for the α-amylase immobilized on chitosan bead (Kumari and Kayastha 2011). Immobilization of maltase on chitin by covalent bonds using glutaraldehyde showed a shift in the optimum pH from 6 to 9 (Habib et al. 2019). As previous reported, a unit change of optimum pH from 5 to 6 was observed after raw starch saccharifying amylase was immobilized on glutaraldehyde functionalized sepa beads (Nwagu et al. 2012a). Immobilized α-amylases ICgAmy1 and ICgAmy2 exhibited the optimum temperature of 70°C as well as free α-amylase CgAmy (Posoongnoen and Thummavongsa 2020). The results can be seen that there was no change in the optimum temperature for ICgAmy1 and ICgAmy2, indicating that there was no enzyme structure change induced by immobilization and the support matrixs. Similar results were obtained for immobilized soybean α-amylase onto chitosan bead, showing the optimum temperature at 70°C, while free α-amylase also showed maximum activity at 70°C (Kumari and Kayastha 2011).

For stability, the α-amylase immobilized on chitosan bead (ICgAmy2) retains over 70% activity at pH 5–9 and up to 90% activity at pH 7. The immobilized α-amylase on DEAE-cellulose (ICgAmy1) retains over 60% activity at pH 5–9 and 86% at pH 7. The enhanced stability of the immobilized α-amylase (ICgAmy1 and ICgAmy2) compared to the free α-amylase (CgAmy), which is stable at pH 6 of 80% activity (Posoongnoen and Thummavongsa 2020), can be suggested to the rigidification of the enzyme structure due to the covalent binding of the enzyme surfaces and support matrix. The enzyme immobilization resulted in increased heat stability with only a 3% loss of activity for ICgAmy2 and 7% loss of activity for ICgAmy1 after 60 min incubation at 70°C. Compared to the free α-amylase of our previous report, the activity was lost by 45% after 60 min incubation at 70°C (Posoongnoen and Thummavongsa 2020). The observed increased stability for ICgAmy1 and ICgAmy2 indicates an enhanced conformational change leading to a loss of enzyme molecular flexibility. These structural changes result in stable enzyme molecules that are less sensitive to changes in environmental variations in temperature. Immobilization of raw starch saccharifying amylase on chitin flakes provides more heat stability. The covalently bound amylase and crosslinked amylase lost less than 5% and 12% of their activity after incubation for 180 min at 60°C, respectively, and the free amylase lost over 50% of activity (Nwagu et al. 2021).

Development of immobilization method of Sword bean α-amylase on DEAE-cellulose and chitosan bead support materials showed novel properties of immobilized enzymes. Both immobilized α-amylase ICgAmy1 and ICgAmy2 had high activity and stability over a wide pH range from pH 5 to 9, which allows them to be used in a variety of industrial applications. The immobilized enzymes ICgAmy1 and ICgAmy2 can be applied in the starch industry, which has a pH value of approximately 6, and can also be used in the detergent industry with a pH in the base range. Comparison with previous studies of wheat α-amylase immobilized on DEAE-cellulose revealed that the immobilized enzyme had an optimum pH of 6. When the pH was increased to 7, 8, and 9, the enzyme activity was reduced to 84, 47, and 22%, respectively (Singh and Kayastha 2014). Furthermore, Sword bean α-amylase also has high activity and stability at high temperatures. At 80°C, the immobilized enzyme activity was as high as 85.5% (ICgAmy1) and 78.4% (ICgAmy2). Importantly, the immobilized enzyme was highly catalytic of 78.3% (ICgAmy2) and 71.5% (ICgAmy1) at temperatures up to 90°C. While wheat α-amylase immobilized on DEAE-cellulose had the highest activity at 70°C. The immobilized enzyme activity decreases to only 72% when the temperature reaches 80°C (Singh and Kayastha 2014).

Compared with the free α-amylase, the K_m_ of immobilized α-amylase ICgAmy1 and ICgAmy2 increased by 2.0 and 2.8 units, respectively after the immobilization process. The increase in K_m_ suggested a low affinity of the immobilized α-amylase toward the substrate compared to the free α-amylase. The change in the K_m_ value of immobilized α-amylases may be due to conformational change in tertiary structure. There was also a steric effect resulting from the restriction of the accessibility of the substrate to the active site of enzyme, which is affected by immobilization. Therefore, the catalytic efficiency was reduced, and increased K_m_ of the immobilized α-amylase (Kumari and Kayastha 2011). The raw starch saccharifying amylases immobilized on chitin flakes by forming covalent bonds and crosslinking showed an increase in K_m_ and lower V_max_ compared to the free enzyme. Immobilization resulted in an increase in diffusional resistance and steric effect, leading to higher K_m_ values. The covalent bond between the enzyme and chitin flakes may cause intense rigidification of the enzyme structure and distortion of some catalytic sites or starch-binding domains on the enzyme molecule which resulted in an increase in the K_m_ value of the immobilized enzyme (Nwagu et al. 2021). Similar changes were also observed in the case of immobilized mung bean α-amylase, which K_m_ was 2.77 mg ml^−1^ for Amberlite and 5 mg ml^−1^ for chitosan bead, approximately higher than the free enzyme 4 times (Tripathi et al. 2007). The immobilized α-amylase from soybean has been reported to increase K_m_ to 4 mg ml^−1^ for chitosan bead and 2.5 mg ml^−1^ for Amberlite (0.71 mg ml^−1^ for Free α-amylase), and there was a decrease in V_max_ to 1.25 µmol min^−1^ mg^−1^ for chitosan beads and Amberlite (2 µmol min^−1^ mg^−1^ for Free α-amylase) (Kumari and Kayastha 2011).

In Sword bean, the α-amylase immobilized on DEAE-cellulose (ICgAmy1) and chitosan bead (ICgAmy2) retained more than 74% residual activity after 10 reuses. While the soybean α-amylase immobilized on chitosan and Amberlite beads had residual activity of 38% and 58%, respectively, after 10 reuses (Kumari and Kayastha 2011). After 10 reuses, raw starch digesting amylase immobilization on glutaraldehyde-activated amberlite beads retained approximately 98% activity (Nwagu et al. 2012b). During the recycling of immobilized α-amylase in catalysis, the enzymes can lose their activity because of denaturation, or the bonds formed during immobilization are weakened, leading to the loss of the enzyme. The high operational stability of the immobilized α-amylase was expected to result from the covalent bonding formed between the glutaraldehyde-activated group on the support materials and the enzyme. This might lead to a stronger quaternary enzyme structure, reducing in enzyme inactivation (Nwagu et al. 2021). Moreover, the storage stability of α-amylase immobilized on DEAE-cellulose (ICgAmy1) and chitosan bead (ICgAmy2) retained up to 80% residual activity after 120 days of storage at 4°C. The residual activity of immobilized soybean α-amylases on chitosan and Amberlite were 60% and 45% after storage for 100 days at 4°C, respectively (Kumari and Kayastha 2011). Immobilization of the enzyme increased operational stability owing to immobilized enzymes can be recovered and re-used in the process.

This research revealed that the α-amylase from Sword bean has been successfully developed to immobilize both DEAE-cellulose and chitosan bead, resulting in the immobilized enzymes of ICgAmy1 and ICgAmy2, respectively. The ICgAmy1 and ICgAmy2 can be applied in industries with high temperatures, such as the starch industry. In the liquefaction process of the starch industry, the temperature is approximately 90°C. In addition, there is a novel point that differs from previous studies of most plant α-amylases which have low activity and stability in base pH range but the α-amylase from immobilized Sword bean (ICgAmy1 and ICgAmy2) had high activity and stability in the base pH range. Therefore, the ICgAmy1 and ICgAmy2 can be applied in industries with basic pH values, such as the highly popular enzyme-based detergent industry.

## Abbreviations

CgAmy: α-amylase from germinating Sword bean seeds
ICgAmy1: immobilized α-amylase from germinating Sword bean seeds on DEAE-cellulose
ICgAmy2: immobilized α-amylase from germinating Sword bean seeds on chitosan beads
